# Educational Technologies

**DOI:** 10.1007/s42010-022-00160-z

**Published:** 2022-11-11

**Authors:** Maren Scheffel, Joachim Wirth

**Affiliations:** Institut für Erziehungswissenschaft, Ruhr-Universität Bochum, Universitätsstraße 150, 44801 Bochum, Deutschland

**Keywords:** Educational Technologies, Technology Acceptance Model, Nutzung von Educational Technologies, Nutzen von Educational Technologies, Educational Technologies, Technology Acceptance Model, Use of Educational Technologies, Benefits of Educational Technologies

## Abstract

Auch wenn der Begriff kein einheitlicher und durch verschiedene Disziplinen geprägter ist, kann unter Educational Technologies der Einsatz (meist computergestützter) Geräte und Medien zum Analysieren, Gestalten und/oder Unterstützen von Lehr- und Lernprozessen subsummiert werden. Dabei sind Educational Technologies sehr vielfältig und können auf den Ebenen des individuellen oder kollaborativen Lernens, des Unterrichts bis hin zur Organisation Schule eingesetzt werden. Ungeachtet der Anstrengungen zum Aufbau einer technischen Infrastruktur an Schulen weisen nationale Bildungsmonitorings darauf hin, dass die wahrgenommene Einfachheit der Nutzung von Educational Technologies dadurch nicht gestiegen ist. Die Beiträge in diesem Themenschwerpunkt zeigen zudem exemplarisch die Vielfalt von Educational Technologies auf. Mit dieser Vielfalt geht einher, dass die Bedingungen, unter denen die jeweilige Technologie den erwünschten Nutzen entfaltet, sehr heterogen sind und generalisierbare Aussagen zum erfolgreichen Einsatz von Educational Technologies nur schwer formuliert werden können. Nichtsdestotrotz erscheinen Forschungsbemühungen im Bereich Educational Technologies lohnenswert, um deren Potenzial für pädagogische und unterrichtliche Prozesse ausschöpfen zu können.

## Abstract (en)

Even if the term is not a uniform one and is characterized by different disciplines, the use of (mostly computer-supported) devices and media for analyzing, designing and/or supporting teaching and learning processes can be subsumed under Educational Technologies. Educational technologies are very diverse and can be used on the levels of individual or collaborative learning, teaching, and even school organization. Despite efforts to build a technical infrastructure in schools, national educational monitoring indicates that the perceived ease of use of educational technologies has not increased as a result. The articles in this issue also illustrate the diversity of educational technologies. This diversity is accompanied by the fact that the conditions under which the respective technology unfolds the desired benefit are very heterogeneous and generalizable statements on the successful use of educational technologies can only be formulated with difficulty. Nevertheless, research efforts in the field of educational technologies seem to be worthwhile in order to exploit their potential for pedagogical and teaching processes.

Unter dem Stichwort der Digitalisierung vollziehen sich an deutschen Schulen seit einigen Jahren, und jüngst verstärkt durch die Herausforderungen, die die COVID 19-Pandemie mit den damit verbundenen Schulschließungen an Schulen stellte, Entwicklungen hin zu einer vermehrten und intensiveren Nutzung von Educational Technologies. Sie betreffen sowohl direkt die Gestaltung des Unterrichts im Klassenzimmer (und im Rahmen des Home Schoolings), aber auch die außerunterrichtliche Kommunikation zwischen Lehrerinnen und Lehrern, Schülerinnen und Schülern, Schulleitungen und Eltern. Bereits vor der Pandemie wurden diese Entwicklungen in Deutschland (mehr oder weniger intensiv) vorangetrieben bspw. durch den Digitalpakt Schule des Bundesministeriums für Bildung und Forschung (BMBF) oder auch durch die Qualitätsoffensive Lehrerbildung des BMBF, in der eine eigene Förderlinie „Digitalisierung“ installiert wurde. Pandemiebedingt erfuhr die Digitalisierung auf materieller Ebene dann in den letzten Jahren auch eine starke Beschleunigung. Doch bereits vor der Pandemie zeigten Heinen und Kerres (2017) auf, dass die empirisch fundierte Entwicklung und Erforschung pädagogischer Konzepte deutlich schleppender vorankommt als die Entwicklung von Hardware und kommerzieller Software. Entsprechend stehen Schulen, Lehrerinnen und Lehrer sowie Schülerinnen und Schüler vor großen Herausforderungen, wenn Educational Technologies professionell in den pädagogischen Kontext einbezogen werden sollen.

Eine dieser Herausforderungen entspringt auch aus dem Umstand, dass schon der Begriff „Educational Technologies“, im Deutschen am ehesten noch mit dem Begriff „Bildungstechnologien“ zu übersetzen, nicht einheitlich definiert ist und somit noch kein geteiltes Verständnis darüber besteht, was zu Educational Technologies zu zählen ist und was nicht. Während der Begriff Educational Technology in der deutschsprachigen Forschungslandschaft erst seit ein paar Jahren auftaucht, ist er im englischsprachigen Raum schon länger in Gebrauch und bildet dort auch eine eigene Disziplin. Spector (2015) beschäftigt sich in seinen „Foundations of Educational Technology“ zunächst mit den Definitionen von technology (die praktische Anwendung von Wissen für einen bestimmten Zweck) und education (der Prozess jemandes Wissen zu verbessern) und definiert dann: „Educational technology involves the disciplined application of knowledge for the purpose of improving learning, instruction, and/or performance“ (Spector 2015, S. 10) durch den Einsatz von technischen Geräten oder Techniken. Ähnlich definieren dies auch Huang et al. (2019, S. 4): „Educational technology refers to the use of tools, technologies, processes, procedures, resources, and strategies to improve learning experiences“. Auch für den deutschen Begriff „Bildungstechnologie“ wird diese Dualität von Gerät/Medium und Prozess in die Definition mit einbezogen: „Während Bildungstechnologie im weiteren Sinn unabhängig von bestimmten Medien und Geräten verstanden wird, verstehen viele die Disziplin in einem engen Sinn als Anwendung von Informations- und Kommunikationstechnik im Bereich der Bildung“ (Niegemann und Weinberger 2020, S. 3). Es wird also betont, dass es bei Bildungstechnologie zwar nicht immer nur um den Einsatz von technischen Medien gehen muss, meist jedoch genau die Verknüpfung von Technologie und Bildungs- bzw. Lernprozess adressiert wird.

Weder in der deutsch- noch der englischsprachigen Forschungslandschaft, die sich mit der Verknüpfung von Technologie und Bildung beschäftigt, sind die beiden Begriffe also so eindeutig definiert, dass Missverständnisse ausgeschlossen sind. Hinzukommt, dass durch die große Interdisziplinarität beteiligter Fachbereiche (z. B. Erziehungswissenschaft, Psychologie, Kognitionswissenschaft, Soziologie, Informatik, etc.) und dem davon abhängigen Fokus der jeweiligen Forschung auch weitere Bezeichnungen möglich und üblich sind: Instructional Technology, Unterrichtstechnologie, Learning Technology, technologie-gestütztes Lernen, Technology-Enhanced Learning, Computer-Assisted Learning, computergestütztes Lernen und Lehren, E‑Learning, etc. Wann genau welcher Begriff erstmalig verwendet wurde, ist nicht mehr wirklich nachzuvollziehen (Niegemann und Weinberger 2020). Gemein ist den Begriffen jedoch, dass (meist computergestützte) Technik im Kontext von Lehr-Lernprozessen eingesetzt wird. Entsprechend verwenden wir im weiteren Verlauf den Begriff Educational Technology und meinen damit den Einsatz (meist computergestützter) Geräte und Medien zum Analysieren, Gestalten und/oder Unterstützen von Lehr- und Lernprozessen.

In Anlehnung an Opfermann et al. (2020) lassen sich drei wichtige Merkmale von Educational Technologies herausstellen: Interaktivität, Adaptivität und Multimedialität. Mit Interaktivität ist hier die Interaktionsbandbreite von Lernenden mit Geräten wie Computern, Tablets oder Smartphones gemeint, die für den Konsum von Lernmaterialien oder das Arbeiten mit Lernsoftware genutzt werden können. Mit Adaptivität ist die Anpassung an individuelle Förderungsbedarfe, Lernziele oder auch Lernverhalten gemeint. Es können also zum Beispiel Lernmengen oder Lernzeiten oder auch Schwierigkeitsgrade von Lernmaterialen so angepasst werden, dass sie auf einzelne Lernende abgestimmt sind. Multimedialität schließlich bezieht sich darauf, dass Lernmaterialen zum Beispiel als Texte, Bild‑, Audio- oder Videodateien vorliegen können und die verschiedenen Darbietungen auch miteinander gemischt und gleichzeitig eingesetzt werden können. Des Weiteren unterscheiden Opfermann et al. (2020) drei Formen von Lehrmedien: Übungssysteme (z. B. Lernprogramme, bei denen es um das Speichern und Abrufen vorhandenen Wissens geht), tutorielle Systeme (z. B. Programme, die durch Unterstützung der Selbsterarbeitung von Inhalten neues Wissen vermitteln) und Simulationssysteme (z. B. Programme, in denen Informationen von Lernenden selbst gesteuert und manipuliert werden). Diese Lehrmedien sind typische Vertreter von Educational Technologies, die gemeinsam haben, dass sie direkt auf den Lernprozess selbst abzielen und die für das Lernen primären Denkprozesse beeinflussen und unterstützen möchten. Neben diesen Lehrmedien gibt es mittlerweile jedoch noch weitere Tools, die zu Educational Technologies zu zählen sind, aber nicht direkt die Informationsverarbeitung der Lernenden adressieren. Beispiele dafür sind Awareness Tools, wie sie zur Unterstützung des Lernens in Gruppen eingesetzt werden (z. B. Bodemer und Schnaubert 2020; Kube et al. in diesem Heft) oder auch Dashboards, die Lehrende über Lernprozesse von Lernenden informieren und dabei unterstützen, Lernenden genau die Hilfe anzubieten, die sie brauchen (z. B. van Leeuwen und Rummel 2019, in diesem Heft).

Unabhängig davon, um welche Art von Educational Technologies es sich handelt, ist ihr erfolgreicher Einsatz abhängig von der Akzeptanz derer, die diese Technologien einsetzen bzw. damit arbeiten und lernen. Damit unterscheiden sich Educational Technologies zwar nicht von jeglicher pädagogisch-didaktischen Methode. Aber gerade in Deutschland hatten es technologiebasierte Ansätze häufig schwer auf Akzeptanz zu stoßen, war die Pädagogik und Erziehungswissenschaft doch lange eher geisteswissenschaftlich ausgerichtet und Technik und Technologien gegenüber eher abgeneigt (Klauer 1973; Niegemann und Weinberger 2020). Diese Skepsis scheint sich auch mindestens bis vor der Pandemie gehalten zu haben. Betrachtet man die Daten der ICILS 2018-Studie, fällt auf, dass an deutschen Schulen Educational Technologies so selten wie in fast keinem anderen teilnehmenden Land zum Einsatz gekommen sind (Schaumburg et al. 2019).

Gemäß des Technology Acceptance Model (TAM) von Davis (1986) hängt die Akzeptanz und damit auch die Nutzung von Technologien maßgeblich vom wahrgenommenen Nutzen sowie von der wahrgenommenen Einfachheit der Nutzung ab. In Bezug auf die technischen Voraussetzungen für eine einfache Nutzung von Educational Technologies sind Schulen in Deutschland nicht zuletzt durch die oben angesprochenen Programme zur Digitalisierung ein gewisses Stück vorangekommen. Es ist mittlerweile keine Besonderheit mehr, wenn Klassenzimmer mit Smartboards und Lehrerinnen und Lehrer mit Tablets ausgestattet sind. Auch sind Tabletklassen oder zumindest Tabletklassensätze zunehmend verbreitet und laut Länderindikator 2021 findet auch der WLAN-Zugriff langsam Einzug in Schulen (Yotyodying und Lorenz 2022). Dadurch werden die technischen Grundvoraussetzungen für die Nutzung von Educational Technologies immer weiter ausgebaut, und man könnte vermuten, dass dadurch auch die wahrgenommene Einfachheit der Nutzung von Educational Technologies erhöht wird. Yotyodying und Lorenz (2022) finden dafür jedoch keinerlei Anzeichen. Die Zufriedenheit der Lehrerinnen und Lehrer mit der IT-Infrastruktur und dem IT-Support ist bis 2021 im Vergleich zu 2017 keinesfalls gestiegen. Dies könnte jedoch auch ein Ergebnis der jetzt vermehrten Nutzung von Educational Technologies sein, die ihrerseits höhere Anforderungen an IT-Infrastruktur und Bedienung stellen. Entsprechend scheint die Herausforderung weiter zu bestehen, Schulen derart auszustatten, dass die Nutzung aktueller Educational Technologies von Lehrkräften als hinreichend einfach wahrgenommen wird. Dafür scheint der rein technische Ausbau der IT-Infrastruktur allein nicht hinreichend zu sein. Die Einführung von Educational Technologies erfordert einen Schulentwicklungsprozess, der Organisations- und Personalentwicklung beinhaltet, alle Akteure von Beginn an einbindet und kontinuierlich evaluiert wird (Eickelmann und Gerick 2017; Wohlfart und Wagner in diesem Heft).

Akzeptanz von Educational Technologies ist nicht nur Resultat der wahrgenommenen Einfachheit ihrer Nutzung, sondern auch ihres wahrgenommenen Nutzens (Davis 1986). Und dieser ist wiederum gekoppelt an Erwartungen und ihre Erfüllung (Wohlfart und Wagner in diesem Heft). Erwartungen an Educational Technologies sind vielfältig wie alleine schon an den in diesem Themenschwerpunkt vorgestellten Educational Technologies deutlich wird. Sie gehen von einem stärkeren Praxisbezug, einer authentischen Darstellung von Lerninhalten und einer hohen Realitätsnähe und Immersion (Götzfried et al. in diesem Heft; Kunz und Zinn in diesem Heft; Nachtigall et al. in diesem Heft) über die Möglichkeit der Interaktion und Anwendung von erworbenem Handlungswissen (Kunz und Zinn in diesem Heft) bis hin zur Unterstützung kollaborativer Lernprozesse (Kube et al. in diesem Heft) oder dem Identifizieren von Unterstützungsbedarfen individueller Lerngruppen (van Leeuwen und Rummel in diesem Heft). Inwiefern und unter welchen Bedingungen diese Erwartungen erfüllt werden, wird in den verschiedenen Beiträgen zu diesem Themenschwerpunkt untersucht. Dabei wird zum einen deutlich, dass die Bewertung des bereits erreichten Nutzens von Educational Technologies und die Bedingungen für ihren erfolgreichen Einsatz auch davon abhängt, wer diese Bewertung durchführt. So weisen Wohlfart und Wagner (in diesem Heft) z. B. darauf hin, dass Personen aus der Schulleitung teilweise zu deutlich positiveren Bewertungen gelangen als Lehrerinnen und Lehrer oder Schülerinnen und Schüler. Erwartungen werden zudem enttäuscht, wenn Educational Technologies weitestgehend in einer Übertragung klassischer Unterrichtseinheiten auf digitale Settings besteht und so kein Gewinn durch den Aufwand, den diese Übertragung erfordert, gesehen wird. Zum anderen zeigen die Studien auf, dass der Nutzen von Educational Technologies von jeweils sehr spezifischen Ausgestaltungen und Bedingungen abhängt, die sich nur schwerlich generalisieren lassen. So zeigt sich z. B. bei Kunz et al. (in diesem Heft), dass die gewünschte Immersion und das Präsenzerleben von Lehramtsstudierenden beim Lernen mit einem 360 Grad-Unterrichtsvideo insbesondere dann auftreten, wenn Lehramtsstudierende bislang noch über wenige praktische Unterrichtserfahrung verfügen. In ihrem Überblicksartikel arbeiten Kube et al. (in diesem Heft) heraus, dass die Computerunterstützung für kooperatives Lernen insbesondere dann wirksam ist, wenn die unterstützten Gruppen eher heterogen zusammengesetzt sind und synchron zusammenarbeiten. Götzfried et al. (in diesem Heft) finden in Bezug auf Podcasts, dass sie insbesondere dann zu Konzeptwechseln führen können, wenn Fachsprache vermieden und Alltagssprache verwendet wird. Und van Leeuwen und Rummel (in diesem Heft) zeigen, dass Lehrerinnen und Lehrer sich vor allen Dingen dann durch Teacher Dashboards unterstützt fühlen, wenn sie unter Zeitdruck arbeiten müssen, was im alltäglichen Unterricht ja nicht zu selten vorkommt. Zusammengenommen zeigen diese Ergebnisse, dass die Voraussetzungen für einen erfolgreichen Einsatz von Educational Technologies so unterschiedlich sind wie die Educational Technologies selbst. Entsprechend besteht für jede einzelne Educational Technology ein hoher Forschungsbedarf, wenn sie pädagogisch sinnvoll zur Analyse, Gestaltung und/oder Unterstützung von Lehr- und Lernprozessen eingesetzt werden soll. Ein Umstand, der den von Heinen und Kerres (2017) formulierten Befund, dass die empirisch fundierte Entwicklung und Erforschung pädagogischer Konzepte deutlich schleppender vorankommt als die Entwicklung von Hardware und kommerzieller Software, teilweise erklären kann.

Zu den Forschungsbedarfen zählt jedoch nicht nur die Untersuchung von optimalen Ausgestaltungen und Einsatzbedingungen von Educational Technologies. Genauso müssen unerwünschte Nebenwirkungen in den Blick genommen werden, um diese möglichst minimieren zu können. So weist die Studie von Nachtigall et al. (in diesem Heft) z. B. darauf hin, dass das durchaus erwünschte Immersionserleben beim Lernen mit 360 Grad-Videos im Fach Geschichte auch dazu führen kann, dass Schülerinnen und Schüler weniger strategisch mit dem Lernmaterial umgehen. Ein explizites Strategietraining kann hier Abhilfe schaffen und Schülerinnen und Schüler dabei unterstützen, das Lernmaterial systematischer zu analysieren und reflektierter damit umzugehen. Kube et al. (in diesem Heft) machen darauf aufmerksam, dass Algorithmen der computerbasierten Unterstützung kollaborativen Lernens u. a. das Risiko beinhalten, Geschlechterstereotype zu reproduzieren statt sie zu mindern bzw. zu verhindern. Sie plädieren entsprechend dafür, Frauen stärker in die Entwicklung solcher computerbasierter Unterstützungssysteme mit einzubeziehen, um diesem Risiko entgegenzuwirken.

Es bedarf einer besonderen unterrichtswissenschaftlichen Forschungsanstrengung, um die Lücke zwischen der technologischen und der pädagogisch-didaktischen Entwicklung im Bereich der Educational Technologies zu schließen. Und es darf bezweifelt werden, dass dies jemals vollständig gelingen wird. Das darf aber nicht davon ablenken, dass der Einsatz von Educational Technologies in Unterricht und Schule ein großes Potenzial bietet. Die Studien in diesem Themenschwerpunkt geben exemplarisch Hinweise darauf, wie diese Potenziale jeweils zu heben sind, so dass Educational Technologies von Nutzen und einfach zu nutzen sind. Und sie zeigen auf, dass sich die Forschungsanstrengungen auszahlen können.
